# Cellular responses to Sindbis virus infection of neural progenitors derived from human embryonic stem cells

**DOI:** 10.1186/1756-0500-7-757

**Published:** 2014-10-24

**Authors:** Jie Xu, Rodney J Nash, Teryl K Frey

**Affiliations:** Department of Biology, Georgia State University, Atlanta, GA USA; Jeevan Bioscience, Inc, Dunwoody, GA USA

**Keywords:** Stem cell infection, Human neural progenitors, Sindbis virus

## Abstract

**Background:**

Sindbis virus (SINV) causes age-dependent encephalitis in mice, and therefore serves as a model to study viral encephalitis. SINV is used as a vector for the delivery of genes into selected neural stem cell lines; however, the toxicity and side effects of this vector have rarely been discussed. In this context, we investigated the cellular responses of human embryonic stem cell (hESCs) derived neural progenitors (hNPCs) to SINV infection by assessing susceptibility of the cells to SINV infection, analyzing the effect of infection on cell proliferation and cell death, and examining the impact of SINV infection on hNPCs markers of stemness.

**Findings:**

We found that hNPCs are highly susceptible to SINV infection. Upon infection, the viruses induced apoptosis to hNPCs while not affecting the expression of cell proliferation markers. Lastly, SINV infections result in significant changes in the expression of key regulators of hNPCs’ plasticity and homeostasis.

**Conclusion:**

The robust and versatile signaling, proliferation, and other cell responses of hESCs-derived hNPCs to virus infection demonstrated that it is a good model to study the pathogenesis of viral-induced neurodevelopmental and degenerative diseases. On the other hand, the toxicity of SINV to hNPCs cells cannot be ignored, and therefore extra care should be taken when using SINV as a vector to deliver genes into human stem cell lines.

## Background

Sindbis virus (SINV), a positive strand RNA virus in the genus *Alphavirus* of the *Togaviridae* family, causes rash and fever in humans and age-dependent encephalitis in mice. The virus has long served as a model to study viral encephalitis induced by viruses in the *Togaviridae* family, as well as other neurovirulent viruses [1, 2]. More importantly, SINV has been widely used to express a variety of genes in cultured neurons and *in vivo* as it provides fast onset and high level of expression of foreign genes [3]. The high and rapid expression of foreign genes from SINV vector is accomplished at the cost of shutting off protein nsP2 synthesis in the infected host cells. As nsP2 are commonly encoded in the SINV vector, whether this machinery leads to the toxicity of the SINV vector in cells are unknown [4]. In recent studies, SINV was used as vector to deliver HIV gp120 into hNPCs and showed a lytic effect to cells, while others using wild-type HIV did not [5, 6]. The mechanisms governing pathogenic outcome and extent of SINV replication in human cells are not well characterized [7–9]. As SINV gains popularity in neurotherapy as an ideal vector for gene transfer into neural stem/progenitor cells, the toxicity, as well as other side effects of these vectors, needs to be addressed [3, 10].

Recently, hNPCs have been developed commercially as a model to study developmental neurotoxicity and neurotherapy [11]. In this study, we used hNP1 cells (ArunA Biomedical), an hNPC line derived from the NIH-approved H9 (WiCell Research Institute’s H9 (WA09)) human embryonic stem cell line [12]. Undifferentiated hNP1 cells stably express the stemness markers Nestin and SOX2, and the cells have the capacity to differentiate into multiple neuronal subtypes, including cholinergic, dopaminergic, and GABAergic neurons, glia, and oligodendrocytes. hNP1 cells have not been immortalized or otherwise transformed, and therefore the potential caveats of transformation are not an issue when using these cells. In addition, hNP1 can grow as a monolayer without fibroblast support, another advantage over other primary neuronal stem cells. Lastly, hNP1 cells have been successfully used in the study of radiation sensitivity, neurotoxicity screening, neurophysiology, tissue engineering, and translational medicine [13–19]. Better still, hNP1 cells have been recently applied to neurotherapies of multiple sclerosis patients; techniques such as SINV-based vector-mediated gene transfer were intensively utilized [20]. Thus, the study of the cellular responses of hNP1 cells to SINV infection will serve as a reference for the application of commercialized hNPCs in stem cell infection and translational medicine field.

Here, we show that hNPCs are highly susceptible to SINV infection with a cytopathic effect (CPE) starting at 24 hours post infection (h.p.i). SINV replicated and disseminated effectively as virus titer increased by 100 fold in hNPC cells, and the percentage of infected cells reached over 85% at later time points. Besides these developments, the virus also triggered apoptosis while not affecting the expression of cell proliferation markers. In addition, reduced expression of hNPCs stemness marker Nestin was observed throughout the infection time course. Close scrutiny suggested that SINV upsets the dedicated balance of hNPC cell signaling, such as STAT3, but not NF-kB and pIRF3. Thus, we added this commercialized hNPC line into another type of human neural stem cells that are susceptible to SINV infection. SINV establish lytic replication cycles in hNPCs, and thus extra care should be taken when using SINV-based vector for gene delivery in stem cell therapy.

## Material and methods

### Cell culture

hNP1™ Neural Progenitor cells (hNPC’s) (ArunA Biomedical) derived from the WA09 hESCs were cultured in Matrigel™ (BD Bioscience) coated tissue culture. Cells were maintained in complete neural expansion medium composed of Neuro-X™ (Jeevan Biosciences, Inc. (Dunwoody, Georgia)) medium supplemented with 20 ng/ml Leukemia Inhibitory Factor (LIF, Millipore), 2 mM L-Glutamine, 0.5 U/ml penicillin, 0.5 U/ml streptomycin (both from Invitrogen), 20 ng/ml basal human fibroblast growth factor (bFGF, Millipore) at 37°C and 5% CO_2_. Culture medium was changed every other day, and hNP1 cells were passaged every 3–4 days using either a cell scraper or manual pipetting. For all the experiments described below, passage 6–10 cells were used. Baby hamster kidney (BHK-21) cells for preparing SINV stock were obtained from the American Type Culture Collection (ATCC) and grown and maintained at 35°C in Dulbecco’s Modified Eagle Medium (D-MEM, Cellgro) with 5% fetal bovine serum (FBS), as described previously [21].

### Virus stock preparation

To prepare SINV stock, 80% confluent BHK cells were infected with the SINV HR strain at a multiplicity of infection (m.o.i) of 0.1. Culture medium was collected at 2 days post infection (d.p.i) when CPE was obvious in the culture. Cell-free (clarified) virus stock was prepared by collecting supernatant of such medium after high speed centrifugation. hNP1 cells were infected as follows: after the removal of culture media, cells were washed once with phosphate buffered saline (PBS) with calcium and magnesium (KCl 2.68 mM, KH_2_PO_4_ 1.47 mM, NaCl 136.89 mM, Na_2_HPO_4_ 8.1 mM, CaCl_2_ 0.9 mM, MgCl_2_ 0.49 mM) and infected with SINV stock at the appropriate m.o.i. The plates were incubated at 37°C for one hour to allow adsorption. The inoculum was removed and replaced by complete neural expansion medium supplemented with growth factors. Culture medium was collected at appropriate times to test for the presence of virus by standard plaque assay on Vero cells.

### SINV growth curve characterization

To characterize SINV growth kinetics, hNP1 cells at 60-70% confluency were infected with SINV at an m.o.i of 1, and the medium was collected at 4, 8, 12, 24, and 48 hours post infection. The amount of infectious SINV in the medium collected from infection hNP1 cells was titered by plaque assay. Plaque assay was performed as previously described with minor modifications [22].

### Immunofluorescent assay (IFA)

Standard immunocyto-fluorescence was performed. hNPCs grown on coated glass-coverslips (80% confluence) were fixed with 4% paraformaldehyde (Electron Microscopy Sciences) in PBS for 10 minutes at room temperature, rinsed twice with PBS and then permeabilized with 0.2% TritonX-100 diluted in PBS for 5–7 min. Primary antibodies used were directed against Nestin (a neural stem marker; 1:450; Neuromics), SINV-NSP (a SINV antigen, 1:100, Eptomics), active-caspase 3 (1:500; Cell signaling), and β-tubulin (cytoskeleton marker, 1:200, Abcam). Secondary antibodies were anti-rabbit/mouse Alexa Fluor 488 and anti-rabbit/mouse Alexa Fluor 594 (1:2000–4000; Molecular Probes-Invitrogen Life Technologies). Fluorescence images were acquired on a Zeiss Axioplan epifluorescence wide-field microscope and processed with AxioVision software. For each condition within the same experiment, at least 3 fields were analyzed. For image quantification, at least three fields in the same experiment were analyzed.

### Flow cytometry

The percentage of cells expressing virus antigens or lineage specific markers was determined by flow cytometry. hNPCs were harvested and washed twice with 2%FBS/PBS (staining buffer) and then fixed by BD Cytofix/Cytoperm solution according to manufacturer’s instruction (BD Bioscience), in aliquots of 100,000 cells in replicate for each antigen. Each aliquot was stained with one or two of the selected cell marker antibodies for 1 hr on ice. Antibodies used were: anti active Caspase-3 (conjugated with V450, BD Pharmingen) and anti-Nestin (conjugated with PE, BD Pharmingen). Cells stained with isotype (mouse IgG V450 or mouse IgG PE, BD Pharmingen) were used as controls. Flow cytometry was performed using a BD LSR Fortessa (BD Bioscience). Data analysis was performed using FACS Diva software (BD Bioscience). The percentage of cells expressing fluorescence intensity greater than the control cells was calculated.

### Proliferation assay

For the assessment of the effect of SINV on hNPCs differentiation, hNP1 cells were seeded onto 60 mm^2^ plates in a density of 2 × 10^6^ cell/plate and infected with SINV at an m.o.i of 1. Mock infected and SINV infected samples were collected at 12, 24, and 48 hours post infection and subjected to an EdU incorporation assay (Invitrogen) by flow cytometry for assessment of cell cycle and proliferation.

### Western blot

Western Blot analysis was performed on cell lysates of hNPCs, either mock infected or SINV infected, at 4, 12, 24, 36, and 48 hours post infection, using protocols described previously [23]. Primary antibodies used were anti-GAPDH (1:5000; Abcam), anti-NF-kB p65 (1:200; Santa Cruz); anti-phospho-STAT3 (1:200; Cell signaling), anti-phospho-IRF3 (1:1000; Eptomics); anti-Nestin (1:500; Neuromics); anti-neuro-filament M (NF-M; 1:500; Neuromics); anti-Tuj1 (1:1000; Abcam); anti-PCNA (1:1000, Santa Cruz) and anti-cleaved-caspase 3 (1:1000; Cell Signaling). For quantification of western blot, films of immunoblot were scanned with a flat-bed scanner, and digital images were imported and quantified using Image J software [24]. Then, the intensities of bands were compared according to their grayscale (http://www.lukemiller.org/journal/2007/08/quantifying-western-blots-without.html).

## Findings

### hNPCs are fully permissive to SINV infection

**Figure 1:**
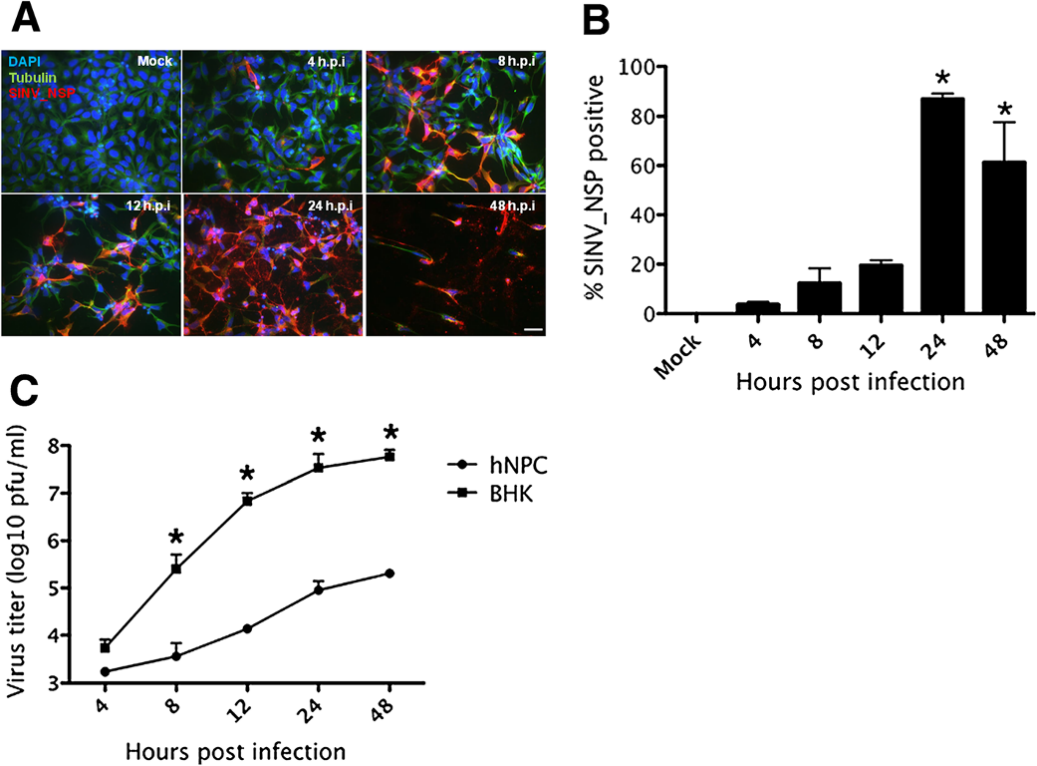
**hNPCs are highly susceptible to SINV infection.** hNPCs were infected with SINV at an m.o.i. of 1 **(A)** At 4, 8, 12, 24, and 48 hours after infection, cells were immunostained with antibodies against tubulin (green) and the SINV nonstructural proteins (SINV_NSP, red). Nuclei were counterstained with DAPI (blue). Bars: 10 um. **(B)** Based on SINV_NSP immune-staining as shown in Figure 1A, the percentage of infected hNPCs was determined at each time point. The results are the means of two independent experiments; at least 200 cells from three different fields were counted at each time point. **(C)** hNPC (hNP1) and BHK cells were infected at an m.o.i of 1. Medium collected at the same time points was titered for infectious virus. Each data point is the average of duplicate titration from three experiments. Error bars indicate SDs. *, statistical significance (p < 0.05) in comparison with the SINV-infected sample of the same time point.

To determine the permissiveness of hNPCs to SINV infection, we plated them onto Matrigel-coated plates and infected them with SINV at an m.o.i of 1. The capacity of the virus to infect, replicate, and disseminate in hNPCs was evaluated by immunofluorescence 4, 8, 12, 24, and 48 h following infection using an antibody directed towards the viral nonstructural protein SINV-NSP. In addition, we used an antibody towards tubulin to monitor cell morphology changes during the infection (Figure 1A). At 4 h following infection, a number of cells, albeit a small number, stained positive for SINV-NSP, revealing their permissiveness to SINV infection. No significant changes in cell shape/morphology were detected at this point. The observation of cultures from 4 to 24 h after infection showed that while only 3.8% ±0.32% of the cells were infected at 4 h.p.i, a large proportion of cells (86.8% ±0.8%) did so by 24 h.p.i (Figure 1B), demonstrating that the virus replicates in hNPCs and disseminates efficiently. CPE (i.e. presence of floaters, elongation of adherent cell bodies) was detected at 24 h.p.i, and at 48 h, few cells remained attached to the plate compared to uninfected controls. The percentage of infected cells no longer increased at 48 h.p.i, probably due to the massive loss of proliferating hNPCs upon SINV infection. Interestingly, elongated cell morphology was noticed in infected cells at this time point, indicating that SINV may induce premature differentiation of hNPCs or otherwise remove the capacity to maintain stemness. Extracellular virus yield was examined as a measurement of SINV replication efficiency (Figure 1C). Virus titers increased by 100 fold from 4 h.p.i (10^3^pfu/ml) to 24 h.p.i (10^5^pfu/ml), at which time the virus had disseminated throughout most of the culture. The viral titer achieved by SINV in hNPCs is 2–3 log lower than those seen in BHK cells (10^7^ or 10^8^ pfu/cell).

### SINV inhibits hNPCs proliferation by inducing apoptosis, but not cell cycle arrest

**Figure 2:**
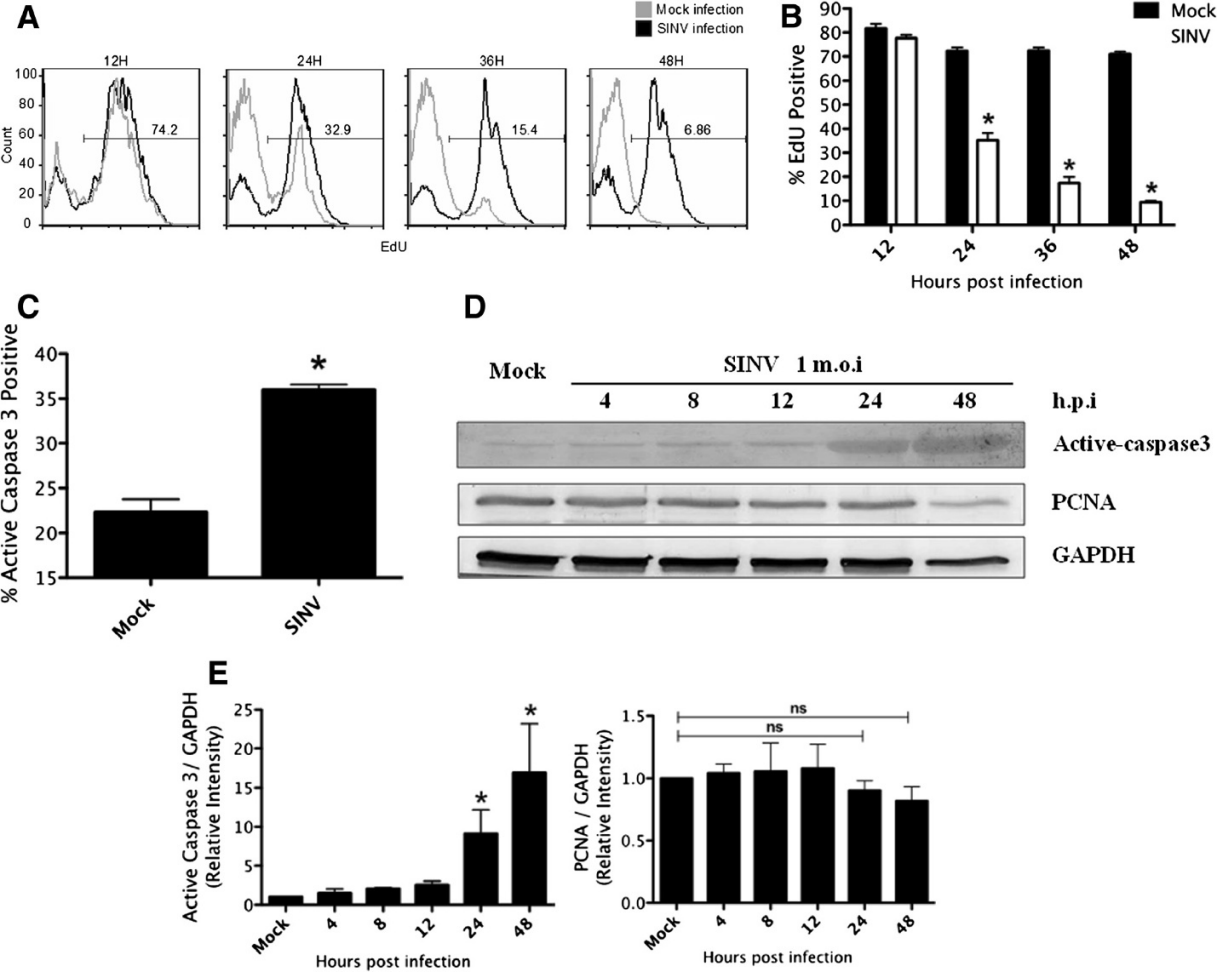
**Effect of SINV infection on hNPCs proliferation and undifferentiated phenotype.** hNPCs were mock infected or infected with SINV at m.o.i 1 **(A, B)** Cell proliferation was analyzed by an EdU incorporation assay. A representative blot is shown in **(A)**, samples were gated on EdU staining positive cells, and percentage of EdU incorporated cells in SINV infected sample were shown. **(B)** Percentage of EdU incorporated cells in mock and SINV infected cells were quantified by flow cytometry. Error bars indicate SDs. *, statistical significance (p < 0.05) in comparison with mock. **(C)** At 48 hour after infection, expression of apoptotic cell marker active caspase 3 was analyzed by flow cytometry. This experiment was repeated at least twice, two titrations per experiment. Error bars indicate SDs. *, statistical significance (p < 0.05) compared to the mock infected control at same time point. **(D, E)** Western Blot analysis of the expression of active caspase 3 and proliferating cell marker PCNA during the infection time course. Each experiment was performed at least three times. A representative blot is shown in **(D)**. Blots were scanned, and relative expression levels of proteins were normalized to GAPDH and shown in **(E)**. Error bars indicated SDs. *, statistical significance (p < 0.05) compared to the mock.

Since SINV led to massive cell loss, we investigated whether the diminished hNPC population was due to SINV-induced cell death or cell cycle arrest. The effect of SINV on hNPC proliferation was quantitatively analyzed by EdU incorporation, which demonstrated a significant reduced cell growth in infected cultures (Figure 2A). At 12 h.p.i, no significant change in EdU labeling was noticed, however, at 24 h.p.i, this reduced drastically from 74.7% ±3.2% in uninfected controls to 33.4% ±1.25% in SINV infected hNPCs (Figure 2B). This negative effect on cell proliferation was not demonstrated until 24 hours after infection, in correlation with virus replication kinetics.

**Figure 3:**
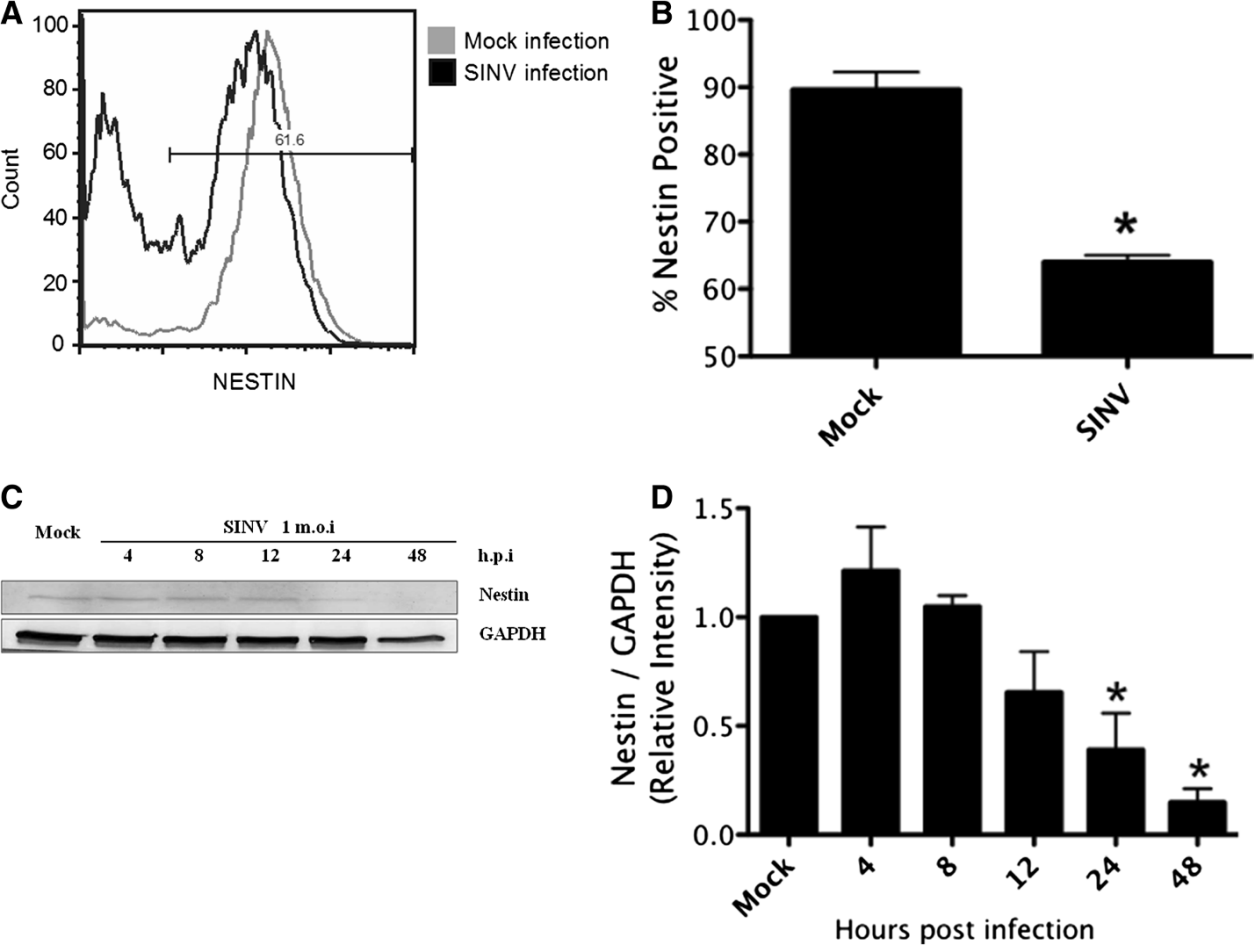
**Effect of SINV infection on hNPCs multipotency/stemness marker Nestin expression.** hNPCs were mock infected or infected with SINV at m.o.i 1 **(A,B)** At 48 hour after infection, expression of stemness marker Nestin was analyzed by flow cytometry. A representative blot is shown in **(A)**. Statistics from three repeats were shown in **(B)**, samples were gated on Nestin staining positive cells, and percentage of Nestin-positive cells in SINV infected sample were shown. Error bars indicate SDs. *, statistical significance (p < 0.05) compared to the mock infected control. **(C,D)** Western Blot analysis of the expression of Nestin during the infection time course. Each experiment was performed at least three times. A representative blot is shown in **(C)**. Blots were scanned, and relative expression levels of proteins were normalized to GAPDH and shown in **(D)**. Error bars indicated SDs. *, statistical significance (p < 0.05) compared to the mock.

To investigate the induction of apoptosis upon SINV infection, at 24 h.p.i mock infected and SINV infected cells were fixed, stained for active-caspase 3, and analyzed by flow cytometry. Compared to uninfected cells, a 2-fold increase in the percentage of active-caspase 3 positive cells in SINV infected culture was observed (Mock 22.5% ± 1.25% vs. SINV infected 35% ± 0.33%) (Figure 3C). To further characterize apoptotic events in hNPCs culture upon SINV infection, the expression of active-caspase 3 was monitored on a protein level by Western Blot throughout the infection time course (Figure 2D). Expression of active-caspase 3 was significantly elevated at 24–48 h.p.i, consistent with our observations of CPE (Figure 2E). This result clearly showed that SINV induces apoptosis, and therefore cell death, in hNPCs.

To address whether SINV mediated growth inhibition in hNPCs results from attenuated cell cycle progression, cell lysates from 4 to 48 h.p.i were used to probe the expression of proliferation cell nuclear antigen (PCNA) by Western Blot (Figure 2D). PCNA is an accessory factor for DNA polymerase δ in eukaryotic cells, and therefore is only expressed in proliferating cells that have robust DNA synthesis. Infections of hNPCs with SINV did not result in significant loss in the expression of PCNA, especially at 24 h.p.i where strong apoptosis was induced (Figure 2E). The reduction seen at 48 h.p.i could possibly be due to massive reduction in cell number, as illustrated by the expression of the internal control GAPDH.

Thus, SINV limited hNPC proliferation primarily by inducing cell death/apoptosis instead of promoting cell cycle arrest.

### Proliferating hNPCs loose cellular multipotency/stemness upon SINV infection

As hNPCs exhibited a neuron-like elongated cell shape at the end of SINV infection time course, we wanted to investigate whether SINV induces premature differentiation or loss of cellular multipotency upon infection. The percentage of Nestin-positive cells was analyzed at 24 hours after SINV infection (Figure 3A). SINV infection decreased Nestin-positive cells by 30% compared to the uninfected control (91% ±1.3% of uninfected vs. 62.2% ±3.33% of SINV infected (Figure 3B). Expression of Nestin was also monitored on the protein level by Western Blot (Figure 3C and D). A similar decrease in Nestin expression was noticed, strongly suggesting that proliferating hNPCs lost their multipotency/stemness during SINV infection. Reduction of Nestin levels was not seen until 24 h.p.i, which is the time point SINV reached its highest infection rate, indicating that alteration in cell multipotency also correlated with virus replication (Figure 3D). To investigate if a premature differentiation was initiated, we probed the cells against lineage specific markers such as Tuj-1, A2B5 and NF-M, however, none of these markers showed a positive staining to hNPCs at 48 h.p.i (data not shown). This suggested that SINV infection impaired hNPCs differentiation potential.

### SINV modulates multiple cell signaling pathways of hNPCs during infection

**Figure 4:**
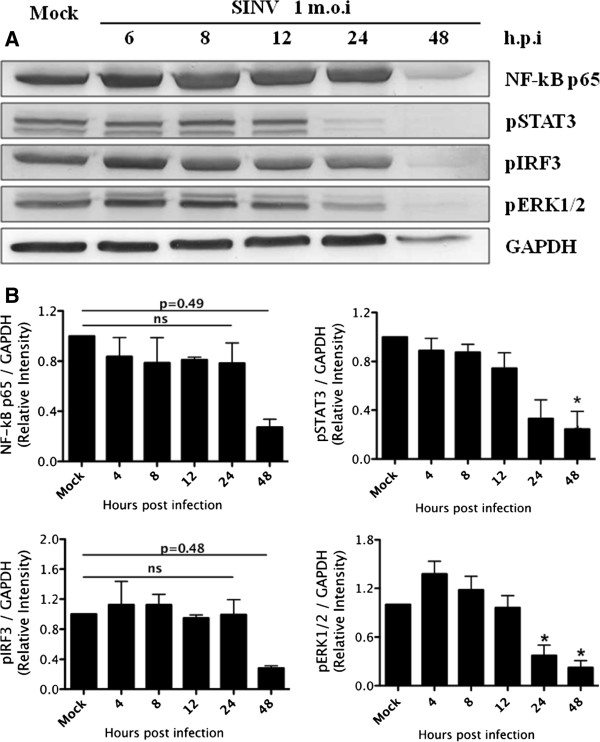
**Western Blot analyzes of the expression of key regulator of differentiation, inflammation responses.** The expression of cellular proteins that regulate key events during differentiation, as well as early inflammation responses, were analyzed during infection time course. Each experiment was performed at least twice. A representative blot was shown. GAPDH: loading control. **(B)** Quantification of western blotting on proteins tested in **(A)**.

Finally, we wanted to investigate the mechanism behind SINV modulation of hNPCs morphology, proliferation and multipotency/stemness. Expression of multiple signaling molecules (NF-kB p65, pSTAT3, pIRF3 and pERK1/2) on crucial regulation pathways during SINV infection course were analyzed by Western Blot (Figure 4A and B). The expression of active phosphorylated (Y705) STAT3 (pSTAT3) was down-regulated by SINV at 24 h.p.i, suggesting a negative regulation of JAK/STAT pathway upon infection. pSTAT3 is required for normal hNPC differentiation, and the reduction in this protein possibly led to impaired differentiation potential [25]. Expression of Phospho-p44/42 MAPK (pERK1/2) was similarly down-regulated by SINV at 24 h.p.i. The MAPK pathway regulates multiple phosphorylation events, including those involved in cell proliferation. Robust expression of pERK1/2 was shown to be pro-survival [26]. Therefore, it is highly possible that SINV impairs hNPC proliferation by repressing pERK1/2 expression, and therefore the regulation of the MAPK pathway. Expression of NF-kB p65 and phospho-interferon regulation factor 3 (pIRF3) were not significantly altered upon SINV infection. As both proteins serve as signaling molecules in IFN induction, it was plausible to suggest SINV did not induce high expression of cytokines, and therefore the inflammatory response. Expression of all four proteins was significantly decreased at 48 h.p.i, possibly due to massive loss of cells at this time point (Figure 4B).

## Discussion

In this study, we addressed the susceptibility of hNPCs to SINV, and their cellular responses to infection. Although SINV does not produce CNS anomalies in human, there are still multiple reasons to study the virus effects on hNPCs: (a) SINV is a model alphavirus that produces age-dependent encephalitis in mice, providing a parallel reference for viral induced human CNS degenerative disease study [27, 28]. (b) SINV is a common test analytic in the class of viral agent for various *in vitro* models [29]. hESC-derived hNPCs are rarely used in viral pathogenesis studies, and another goal of this study is to assess the feasibility of hNPCs as a model to study viral infection. (c) SINV-based vectors have been widely employed in stem cell infection and tumor therapies in human species, however, little is known about the toxicity of the vector itself. Therefore, in the current study, we evaluate the potential effect of using SINV as vector in the studies of human cells as well as in gene therapies of cancer [30]. For the first time in the Alphavirus genus, we report that SINV infects hNP1 cells and significantly attenuates cell proliferation. The virus induces robust cell death and abruptly terminates hNPC multipotency. The result of our studies could shed light on multiple aspects discussed above.

We have shown that hNPCs are fully permissive for SINV infection. The virus is able to establish productive replication and dissemination in culture, since over 90% of cells were infected at the time peak virus titer achieved, which corresponds to its neurotropic nature. Highly efficient cell entry of SINV is probably due to the abundant availability of the cell surface receptor, laminin, on hNPCs, which has been shown as a receptor for SINV in mammalian cells [31]. SINV infection of human cells has not been extensively studied; however, there are several reports that human brain cells are susceptible to SINV infection. In a comparison of oncolytic potential with eight other viruses, SINV was shown to infect nearly 100% of human glioblastoma cells (U-87MG) and induce apoptosis immediately upon its entry. The spread of SINV has also been demonstrated in human brain microvascular endothelial cells (HBMECs) where the virus infection renders cells hypersensitive to the inflammatory inducer Bradykinin [9, 32]. Here, we also demonstrated sufficient virus replication of SINV in hNPCs and its potential to deplete said cell pool. Therefore, it is intriguing why the virus is not neurovirulent in human CNS. SINV has also been shown to replicate in human peripheral mononuclear cells (PBMC) and decrease cell adhesion [29]. Thus, it is highly possible that the virus is eliminated by the immune system before entering the CNS, as the virus was shown to enter the brain through hematogenous route in mice model [1].

Several viruses actively induce apoptosis at late stages of infection, thus allowing the dissemination of progeny viruses, while avoiding host inflammatory and immune responses [33]. In mice models, NPCs are very susceptible to SINV induced apoptosis as early as one day post infection [29]. It has been recently shown that Coxsackie virus B (CVB3) and Cytomegalovirus (CMV) can both induce apoptosis in hNPCs, and thus achieve optimal viral dissemination [34, 35]. Consistent with these reports, we observed a strong induction of apoptosis in SINV-infected hNPCs, as illustrated by an increase in the active-caspase 3 positive cell population and protein expression at late stage of infection. Further validation of proliferation damage in hNPCs with progressive infection was obtained by EdU incorporation analysis, which clearly demonstrated that SINV impacted cell metabolism since there was a significant decrease in EdU uptake compared to the uninfected control. Interestingly, we did not observe any reduction of cell cycle progression in hNPCs as the relative expression of PCNA was consistent even at a later time points of infection, indicating that cell cycle arrest does not contribute to the reduced NPC population following infection. Thus, considering similarities between human NPC and murine NPC in their responses to SINV infection, our study validated that hNP1 cells, as an established cell line, retained their capacities to respond effectively and efficiently, and generated genuine response to SINV infection.

Next, we sought to determine if SINV impacts differentiation of hNPCs. However, as the virus almost depleted the hNPC pools in 2 days, it is thus impossible to initiate any type of differentiation in presence of the virus. But still, we were able to capture a significant reduction of stemness marker Nestin expression in proliferation hNPCs after infection, indicating the virus infection triggered abnormal neural precursor development. Reduced Nestin expression has been highlighted in a recent study in which the researchers reported that a SINV-based vector expressing HIV envelope protein (SIN-HIVenv) could impair murine neural stem cell survival and expression of Nestin [6]. The role of HIVenv in damaging NPCs was ruled out by other groups, as it was reported that few apoptosis or TUNNEL positive NPCs were detected following exposure of the cells to either high concentration of gp120, or in the SGZ of gp120 transgenic mice [36, 37]. Thus, it is the SINV vector that disturbs Nestin expression observed in the neural stem cells. Taking these studies together with ours, we showed that SINV impacts the multipotency of NPCs, and therefore extra care should be taken when using SINV-based vector to deliver genes into NPCs. Further studies should focus on determining if the reduced expression of Nestin is directly triggered by SINV, or by a bystander effect, as well as characterizing the cell lineage that hNPCs tend to differentiate into after SINV infection.

In the last part of our study, we took advantage of the strong cell responses that SINV triggered to investigate cell-signaling profile of hNPCs, which regulated its diverse response to pathogens.

The JAK/STAT pathway plays a pivotal role in balancing hNPCs lineage specific differentiation [25]. The reduced pSTAT3 level in SINV-infected hNCPs indicates that the virus probably impaired cell specific differentiation into this cell type. Extracellular signal-regulated kinases (ERK1/2) regulate multiple cell events, including apoptosis in hNPCs [26, 32]. In our study, we observed that SINV infection induced the phosphorylation of both ERKs at early time points post infection. The level of pERK1/2 was decreased later on, suggesting that the virus interplays with cellular apoptosis signaling with progression of infection, possibly to achieve its optimal replication and dissemination in culture. Consistent with our study, these changes of ERK1/2 expression upon SINV infection were also reported in HMBCs [38]. NF-kB plays a central role in cellular stress responses and in inflammation by controlling the expression of a network of inducers and effectors; in this way, it defines the response to a specific pathogen [39, 40]. Surprisingly, our results showed that expression of NF-kB was not altered by SINV infection, an indication of a lack of an inflammatory response during infection. IRF3 mediates Type I-IFN induction and signaling. Type-I IFN signaling was enhanced during hNPCs differentiation, suggesting maturation of the innate immune system during fetal development [41]. However, in our study, we did not observe any changes of IRF3 induction upon virus infection, implying a lack of IFN responses to SINV infection. On the other hand, we may have simply verified the immaturity of innate immune system in hNPCs [41].

In summary, we showed that SINV establishes productive infection to hNPCs, induces massive apoptosis/cell death, and alters stem cell marker expression. This is in combination with the virus’ ability to avoid cell inflammation responses by upsetting specific cell signaling and taking advantage of hNPCs’ intrinsic immaturity in innate immune system. In particular, the latter may contribute to the age dependence of neurological disease seen in the SINV-mouse model. The robust and diverse cell responses of hESC-derived hNPCs to SINV infection demonstrated that it is a good model to study stem cell infection. We are adding hNP1 cells to a novel type of human stem cells that are susceptible to SINV infection, and prove its cytopathic effect in stem cell lineages. Therefore, extra care should be taken when utilizing SINV-based vectors for human disease gene therapy and stem cell infection.

## Authors’ information

JX is a Postdoctoral Fellow in Department of Pathobiology, University of Pennsylvania School of Veterinary Medicine.
